# Loneliness, social isolation, and health-related quality of life in older adults

**DOI:** 10.1007/s10433-025-00905-6

**Published:** 2026-02-16

**Authors:** Marcela Durán-Arias, Humberto Yévenes-Briones, José R. Banegas, Fernando Rodríguez-Artalejo, Esther Lopez-Garcia, Francisco Félix Caballero

**Affiliations:** 1 Faculty of Medicine. Department of Preventive Medicine and Public Health, Universidad Autónoma de Madrid, Arzobispo Morcillo, 4, 28029 Madrid, Spain; 2 Centro de Investigación Biomédica en Red de Epidemiología y Salud Pública (CIBERESP), Instituto de Salud Carlos III, Madrid, Spain; 3 IMDEA-Food Institute, CEI UAM+CSIC, Madrid, Spain

**Keywords:** Social variables, Loneliness, Social isolation, Physical health-related quality of life, Mental health-related quality of life

## Abstract

Social variables have been associated with health status, especially in older people. However, it is unknown how loneliness and social isolation are differentially associated with physical and mental components of health-related quality of life (HRQoL). We aimed to examine this association, overall and by sex. We used data obtained from 1808 community-dwelling individuals older than 65 years participating in the Seniors-ENRICA 2 cohort. Baseline phase was conducted between December 2015 and June 2017, and then a two-year follow-up period was considered. Loneliness was assessed using the three-item UCLA Loneliness Scale, while social isolation was measured using a four-component index, which included contact with family members and friends. HRQoL was assessed with the SF-12 questionnaire, using its physical and mental components. Multivariable linear regression models were used in the overall sample and stratified by sex. Higher scores in loneliness were associated with a decrease in the physical HRQoL [Coef. =  − 0.31, 95% confidence interval = (− 0.60, − 0.01), *p* = 0.042] and mental HRQoL [− 0.76 (− 1.11, − 0.43), *p* < 0.001]. There was no significant association between social isolation and HRQoL. The association between loneliness and the physical HRQoL was different by sex (*p* for interaction term = 0.028), and higher levels of loneliness were related to lower physical HRQoL scores only in women [− 0.53 (− 0.92, − 0.14), *p* = 0.007]. Loneliness had an independent association with HRQoL. This relationship seems to be stronger in women, which highlights the importance of a sex perspective in interventions to improve the HRQoL.

**Supplementary Information:**

The online version contains supplementary material available at 10.1007/s10433-025-00905-6.

## Introduction

Population aging is an increasing trend around the world. The last decades have seen an increase in life expectancy and better survival rates (Dlima et al. 2024; Rudnicka et al. 2020). By 2022, the percentage of older people (65 years and above) was between 10 and 20% in most countries, and this is higher in high-income countries (United Nations 2023). Projections indicate that, by 2037, 26% of the worldwide population will be 65 years and over (Rudnicka et al. 2020). Accordingly, the importance of a healthy aging process with optimal physical and mental well-being is highlighted; this includes, among others, social factors like high quality of social connections (Casey and Seidman 2022; Rico-Uribe et al. 2016; World Health Organization 2017, 2023a).

Among the most studied social factors related to health are socioeconomic status, educational level, eating habits, and healthy lifestyles, but social isolation and loneliness are also key factors that can be positive or negative for health, as there is evidence that when the social networks are weakened there may be some deterioration in health and quality of life, including depressive episodes, dementia, increased cardiovascular risk, and mortality in general (Braveman and Gottlieb 2014; United Nations 2023; Valtorta et al. 2018). On the other hand, a strong social network could facilitate more activities outside the house that can also help to increase the network and reduce sedentary lifestyles and their associated risks (Pollak et al. 2024; Tyler et al. 2020). However, if social networks are strong and allow for considerable social support, this could reduce loneliness and social isolation, and the benefits could have a positive impact on quality of life in older people (Dahlberg et al. 2022; Yu et al. 2021).

Social isolation is related to the objective aspects of isolation, such as living alone, having few contacts, or little participation in different social activities (Holt-Lunstad et al. 2015). Loneliness refers to the perception or subjective aspect between existing and desired social relationships (Shankar et al. 2011). Although social isolation and loneliness could be associated, they are independent concepts and their effect on health-related outcomes should be separately studied (Freak-Poli et al. 2022; Schrempft et al. 2019). Health-related quality of life (HRQoL) is the general perception of the impact of health status on well-being and quality of life. This concept can be applied to both healthy individuals and people with a medical condition (Mielenz et al. 2006; Yin et al. 2016).

The interaction between loneliness and social isolation has been widely studied with different procedures and different populations, but their association with HRQoL is not well known, especially among older population (Hong et al. 2023; Leigh-Hunt et al. 2017). Moreover, the assessment of differential associations by sex is especially important, since men and women often experience loneliness, social isolation, and HRQoL in a different way (Coley et al. 2017; Nicolaisen and Thorsen 2014).

There is a need to disentangle how social factors are related to HRQoL in aging population, as understanding these dynamics, which can evolve differently over time, is essential for developing targeted interventions that promote healthy aging. We hypothesize that both loneliness and social isolation will have an inverse association with HRQoL, and these social factors could have a differential association with physical and mental HRQoL. Therefore, this study examines the association between loneliness and social isolation, and the physical and mental components of HRQoL among a relatively large sample of community living older individuals. Potentially different associations by sex are also assessed to identify whether the main study results vary between men and women.

## Material and methods

### Study design

This study includes data from the Seniors-ENRICA 2 cohort (Estrada-De León et al. 2021; Ortolá et al. 2022). The baseline phase of this cohort was recruited between December 2, 2015, and June 23, 2017, by sex- and district-stratified random sampling. A total of 3273 community-dwelling individuals of 65 years old and above who lived in Madrid and 4 nearby towns (Getafe, Torrejón de Ardoz, Alcalá de Henares, and Alcorcón) participated in the baseline phase. A follow-up phase was conducted between September 25, 2018, and October 29, 2019. From the 3273 participants initially interviewed, a total of 46 died before being conducted the follow-up phase and 1333 did not participate. A total of 1808 individuals, with complete data in the study variables, comprised the analytical sample. This analytical sample had a mean follow-up period of 2.35 years (858 days). Data were collected using a telephone interview and two subsequent home visits to perform a physical examination, take blood samples and fill out the dietary history. The instruments, procedures used for data collection, and study design have been described elsewhere (Rodríguez-Artalejo et al. 2011). Written informed consent was obtained from all participants, and the study protocol was approved by the Clinical Research Ethics Committee of La Paz University Hospital (Madrid, Spain) (PI-1793 -31 October 2014, PI-3554 12 January 2012).

### Study variables

Loneliness was assessed using the Three-Item UCLA Loneliness Scale (Hughes et al. 2004) that includes the three following questions: How often do you feel that you lack companionship? How often do you feel left out? How often do you feel isolated from others? The possible answers were: 1 = hardly ever; 2 = some of the time; 3 = often. Then, a new variable was created with the total number of these previous variable answers, obtaining a continuous scale from 3 to 9, where higher values indicated higher levels of loneliness. Cronbach’s alpha value obtained for this instrument was 0.82 for the analytical sample considered at baseline in our study.

Social isolation score was calculated based on the following four components: (1) not living with a partner; (2) having less than weekly contact with family members (other than those living with the participant); (3) having less than weekly contact with friends or neighbors; and (4) not attending any organization, senior clubs, or religious services. The presence of each component was scored as 1, resulting in a total value ranging from 0 to 4, with higher scores indicating a greater degree of social isolation (Shankar et al. 2011).

HRQoL was assessed using the SF-12 health questionnaire (Ware Jr et al. 1996) (Supplementary Table S1), which comprises twelve questions that assess physical and mental health components (physical and mental HRQoL, respectively), including physical functioning, physical health problems, bodily pain, general health, energy and feelings of fatigue, emotional problems, and mental health items. Each of the 12 questions has from 2 up to 5 possible answers options. For example, the second question: “Does your health now limit you in moderate activities (e.g., moving a table, pushing a vacuum cleaner, bowling)?” has three possible answers that include “Yes, limited a lot; yes, limited a little; No, it does not.”.

HRQoL was assessed at baseline and after the follow-up period. In our study, the Cronbach's alpha value obtained for the SF-12 was 0.88 in both baseline and follow-up phases. Considering the study setting, two different scores for both physical and mental HRQoL were obtained by means of a weighted sum, since each of the answers to the SF-12 items had a weight assigned to physical HRQoL and another one to mental HRQoL. These weights are shown in Supplementary Table S1 and a detailed description on how to obtain them in a Spanish sample is described elsewhere (Vilagut et al. 2008). Scores in both physical and mental HRQoL ranged between 0 and 100, with higher scores indicating a better perception of quality of life related to physical and mental health, respectively.

### Covariates

Sociodemographic data were collected through interviews, where participants were asked for sex, age (in years), and educational attainment (primary school, high school, and university studies). Weight and height were measured in standardized conditions, and the body mass index (BMI) was calculated as weight (kg) divided by squared height (m^2^). Three categories for BMI were considered in the analysis: < 25 (normal weight), 25–29.9 (overweight), and ≥ 30 kg/m^2^ (obesity) (World Health Organization 2000). Tobacco consumption was self-reported, and participants were categorized as “never smokers”, “former smokers”, and “current smokers”.

Physical activity was assessed with the validated questionnaire developed in the European Prospective Investigation into Cancer and Nutrition (EPIC) cohort study in Spain (Pols et al. 1997). Participants were asked how many hours they spent in a typical week during the last year on walking, cycling, gardening, do-it-yourself (DIY) activities at home, playing sports, and climbing stairs. Tertiles of physical activity (in metabolic equivalent tasks, h/week) were derived for the analysis.

Finally, baseline chronic conditions were self-reported with the specific question: “has your doctor told you if you currently have, or have you had in the last year, any of the following diseases?” Participants were asked for neurological diseases (having Parkinson’s disease, Alzheimer’s disease, and/or dementia), cardiovascular diseases (having acute myocardial infarction, stroke, congestive heart failure, and/or atrial fibrillation), arterial hypertension, and diabetes (by the question “Has your doctor ever told if you have high blood sugar?”).

### Statistical analysis

Sociodemographic and clinical variables were described at baseline. Potential differences by sex in sociodemographic variables and comorbidities were assessed using the unpaired Student t test for quantitative variables and the Chi-square test for the categorical ones. An attrition analysis was conducted for assessing potential differences in sociodemographics and clinical characteristics at baseline between the 1808 participants who comprised the analytical sample and the 1379 ones who did not participate in the follow-up phase.

Multivariable linear regression models were used to assess the association between social variables (loneliness and social isolation) measured at baseline and the scores in physical and mental HRQoL obtained from the SF-12 questionnaire. Since loneliness and social isolation are two independent concepts (Freak-Poli et al. 2022) and the Pearson correlation coefficient between both variables was found to be moderately low in our study [*r* = 0.19, 95% CI = (0.16, 0.22)], they were analyzed simultaneously as independent variables and their relationships with HRQoL were adjusted for the covariates considered. Potential associations were expressed as the non-standardized regression coefficient and a 95% confidence interval (CI). Standardized coefficients (β) were also provided as effect size measures as they can be used to assess which variables have the highest association with the outcome variable. The goodness of fit of the linear regression models was assessed by means of the adjusted R^2^.

A sequential approach in the multivariable linear regression models was considered for assessing the relationships between social factors and physical and mental HRQoL. Three nested models were built progressively for considering sociodemographic characteristics, lifestyle behaviors, and the presence of chronic conditions as potential confounders. Specifically, the first model was adjusted for sex, age, educational attainment, and the baseline status of the dependent variable (physical or mental HRQoL, whatever corresponds); the second one was additionally adjusted for BMI, tobacco consumption, and physical activity; and finally, the third one was also adjusted for the presence of cardiovascular diseases, neurological diseases, hypertension, and diabetes.

The analyses were stratified by sex to evaluate potential differences in the associations between social variables and HRQoL in men and women separately. Interaction terms for social variables (loneliness and social isolation) by sex were included in the corresponding models in the overall sample. This analysis was conducted because higher levels of loneliness have been found for women in previous studies (Boehlen et al. 2022; Nicolaisen and Thorsen 2014).

Statistical analysis was performed using Stata software (version 17). All results with a *p*-value < 0.05 were considered statistically significant.

## Results

Table 1 shows the baseline sociodemographic characteristics of participants by sex. About 51% of participants were women, and the overall mean age was 71.4 years old (SD = 4.2). Almost 60% of participants had primary studies or a lower educational level, but this percentage was higher in women (67.9%) than in men (51.3%); however, the percentage of people with university studies was clearly higher in men (27.6% vs. 14.6%) (*p* < 0.001). In terms of the baseline HRQoL score, men had a higher score than women in both physical and mental HRQoL (p < 0.001). Regarding the social isolation score, significant differences were not found by sex, while the loneliness score was significantly higher in women (*p* < 0.001).

**Table 1:** Sociodemographic and clinical characteristics by sex at baseline

	Overall (*n* = 1808)	Men (*n* = 885)	Women (*n* = 923)	p*
Physical HRQoL, Mean ± SD	51.1 ± 9.4	53.1 ± 7.7	49.2 ± 10.4	< 0.001
Mental HRQoL, Mean ± SD	50.9 ± 9.1	53.3 ± 6.8	48.6 ± 10.4	< 0.001
Social isolation, Mean ± SD	0.8 ± 0.8	0.8 ± 0.8	0.8 ± 0.8	0.62
Loneliness, Mean ± SD	3.7 ± 1.4	3.4 ± 1.2	3.9 ± 1.5	< 0.001
Age (years), Mean ± SD	71.4 ± 4.2	71.1 ± 4.1	71.7 ± 4.3	0.002
Educational attainment, *n* (%)				< 0.001
Primary school or lower	1081 (59.8)	454 (51.3)	627 (67.9)	
Secondary school	348 (19.3)	187 (21.1)	161 (17.4)	
University studies	379 (21.0)	244 (27.6)	135 (14.6)	
Body mass index, n (%)				< 0.001
< 25 kg/m^2^	487 (26.9)	206 (23.3)	281 (30.4)	
25–29.9 kg/m^2^	859 (47.5)	461 (52.1)	398 (43.1)	
≥ 30 kg/m^2^	462 (25.6)	218 (24.6)	244 (26.4)	
Smoking status, n (%)				< 0.001
Never smoker	930 (51.4)	275 (31.1)	655 (71.0)	
Former smoker	714 (39.5)	509 (57.5)	205 (22.2)	
Current smoker	164 (9.1)	101 (11.4)	63 (6.8)	
Chronic conditions, n (%)				
Cardiovascular diseases	117 (6.5)	55 (6.2)	62 (6.7)	0.66
Neurological diseases	217 (12.0)	44 (5.0)	173 (18.7)	< 0.001
Hypertension	993 (54.9)	490 (55.4)	503 (54.5)	0.71
Diabetes	305 (16.9)	166 (18.8)	139 (15.1)	0.036

SD = standard deviation; HRQoL = health-related quality of life; * Unpaired t test was used for comparing means in quantitative variables by sex, while Chi-square test was used for comparing percentages in categorical variables by sex

When assessing differences in sociodemographic characteristics at baseline between the analytical sample and those who did not participate in the follow-up phase, the mean age was higher in those who participated only at baseline (72.4 ± 4.7 years vs. 71.4 ± 4.2 years, *p* < 0.001). The percentage of women (55.9% vs. 51.1%, *p* = 0.006) and people with primary studies or lower (70.5% vs. 59.8%, *p* < 0.001) was also higher in this group. The attrition analysis for the sociodemographic and clinical characteristics considered in this study is shown as supplementary material (Supplementary Table S2).

After two years of follow-up, social isolation did not show a significant association with the physical HRQoL [Coef. = 0.11, 95% CI = (− 0.37, 0.59), *p* = 0.65] (Table 2). On the other hand, those with higher scores in loneliness showed lower scores in physical HRQoL [− 0.31 (− 0.60, − 0.01), *p* = 0.042] in the two-year follow-up period, after adjusting for sociodemographic characteristics, lifestyle behaviors, and the presence of several chronic conditions.

**Table 2:** Multivariable linear regression model for the association between social isolation and loneliness, and physical health-related quality of life, after the two-year follow-up period

	Model 1			Model 2			Model 3		
	Coef. (95% CI)	|β|	*p*	Coef. (95% CI)	|β|	*p*	Coef. (95% CI)	|β|	*p*
Social isolation	0.18 (− 0.30, 0.67)	0.01	0.46	0.19 (− 0.29, 0.67)	0.02	0.44	0.11 (− 0.37, 0.59)	0.01	0.65
Loneliness	− 0.45 (− 0.74, − 0.16)	0.06	0.002	− 0.47 (− 0.76, − 0.17)	0.06	0.002	− 0.31 (− 0.60, − 0.01)	0.04	0.042
Sex (Ref. Man)	− 1.79 (− 2.60, − 0.99)	0.09	< 0.001	− 1.75 (− 2.62, − 0.87)	0.09	< 0.001	− 1.65 (− 2.53, − 0.77)	0.08	< 0.001
Age, years	− 0.21 (− 0.31, − 0.12)	0.09	< 0.001	− 0.21 (− 0.30, − 0.12)	0.09	< 0.001	− 0.22 (− 0.31, − 0.13)	0.09	< 0.001
Educational level (Ref. Primary school or lower)									
Secondary school	0.36 (− 0.65, 1.37)	0.01	0.48	0.29 (− 0.72, 1.30)	0.01	0.58	0.15 (− 0.84, 1.15)	0.01	0.76
University studies	1.01 (0.02, 2.00)	0.04	0.046	0.94 (− 0.06, 1.94)	0.04	0.06	0.89 (− 0.10, 1.87)	0.04	0.08
Body mass index (Ref. < 25 kg/m^2^)									
25–29.9 kg/m^2^				− 0.99 (− 1.92, − 0.07)	0.05	0.036	− 0.78 (− 1.71, 0.15)	0.04	0.10
≥ 30 kg/m^2^				− 2.86 (− 3.93, − 1.78)	0.13	< 0.001	− 2.30 (− 3.40, − 1.20)	0.10	< 0.001
Tobacco consumption (Ref. Never smoker)									
Former smoker				− 0.20 (− 1.09, 0.68)	0.01	0.65	− 0.10 (− 0.98, 0.78)	0.01	0.82
Current smoker				0.12 (− 1.29, 1.53)	0.00	0.87	− 0.01 (− 1.41, 1.39)	0.00	0.99
Physical activity (Ref. 1st tertile)									
2nd tertile				0. 32 (− 0.64, 1.29)	0.02	0.51	0.13 (− 0.82, 1.09)	0.01	0.79
3rd tertile				1.41 (0.38, 2.44)	0.07	0.007	1.12 (0.09, 2.14)	0.05	0.032
Cardiovascular diseases (Ref. No)							− 3.49 (− 5.06, − 1.92)	0.09	< 0.001
Neurological diseases (Ref. No)							− 2.30 (− 3.53, − 1.08)	0.08	< 0.001
Hypertension (Ref. No)							− 0.65 (− 1.44, 0.14)	0.03	0.11
Diabetes (Ref. No)							− 1.27 (− 2.30, − 0.23)	0.05	0.017
Adjusted R^2^	0.30			0.32			0.33		

Model 1 was adjusted for sex, age, educational attainment, and physical health-related quality of life at baseline; Model 2 was additionally adjusted for body mass index, tobacco consumption, and physical activity; Model 3 was additionally adjusted for the presence of chronic conditions (cardiovascular diseases, neurological diseases, hypertension, and diabetes)

After adjusting for covariates, those with higher levels of loneliness showed lower scores in mental HRQoL [− 0.76 (− 1.11, − 0.43), *p* < 0.001] (Table 3). The association between social isolation and mental HRQoL was not found to be significant [0.31 (− 0.21, 0.82), *p* = 0.24].

**Table 3:** Multivariable linear regression model for the association between social isolation and loneliness, and mental health-related quality of life, after the two-year follow-up period

	Model 1			Model 2			Model 3		
	Coef. (95% CI)	|β|	*p*	Coef. (95% CI)	|β|	*p*	Coef. (95% CI)	|β|	*p*
Social isolation	0.33 (− 0.18, 0.84)	0.03	0.20	0.36 (− 0.15, 0.87)	0.03	0.17	0.31 (− 0.21, 0.82)	0.02	0.24
Loneliness	− 0.85 (− 1.18, − 0.52)	0.12	< 0.001	− 0.84 (− 1.17, − 0.50)	0.12	< 0.001	− 0.76 (− 1.11, − 0.43)	0.11	< 0.001
Sex (Ref. Man)	− 2.42 (− 3.28, − 1.57)	0.12	< 0.001	− 2.48 (− 3.42, − 1.55)	0.12	< 0.001	− 2.31 (− 3.25, − 1.37)	0.12	< 0.001
Age, years	− 0.12 (− 0.21, − 0.02)	0.05	0.019	− 0.12 (− 0.22, − 0.02)	0.05	0.017	− 0.13 (− 0.23, − 0.03)	0.06	0.010
Education level (Ref. Primary school or lower)									
Secondary school	− 0.03 (− 1.10, 1.04)	0.00	0.96	− 0.07 (− 1.15, 1.00)	0.00	0.89	− 0.09 (− 1.17, 0.98)	0.00	0.86
University studies	0.39 (− 0.65, 1.44)	0.02	0.46	0.39 (− 0.67, 1.45)	0.02	0.47	0.38 (− 0.68, 1.44)	0.02	0.49
Body mass index (Ref. < 25 kg/m^2^)									
25–29.9 kg/m^2^				0.46 (− 0.53, 1.45)	0.02	0.36	0.54 (− 0.45, 1.54)	0.03	0.29
≥ 30 kg/m^2^				− 0.36 (− 1.50, 0.77)	0.02	0.53	− 0.09 (− 1.27, 1.08)	0.00	0.88
Tobacco consumption (Ref. Never smoker)									
Former smoker				− 0.14 (− 1.09, 0.81)	0.01	0.77	− 0.12 (− 1.07, 0.83)	0.01	0.80
Current smoker				− 0.94 (− 2.44, 0.57)	0.03	0.22	− 1.00 (− 2.51, 0.50)	0.03	0.19
Physical activity (Ref. 1st tertile)									
2nd tertile				0.56 (− 0.46, 1.58)	0.03	0.28	0.48 (− 0.54, 1.51)	0.02	0.35
3rd tertile				0.11 (− 0.98, 1.19)	0.00	0.85	− 0.02 (− 1.11, 1.07)	0.00	0.97
Cardiovascular diseases (Ref. No)							− 0.53 (− 2.20, 1.14)	0.01	0.53
Neurological diseases (Ref. No)							− 2.46 (− 3.81, − 1.11)	0.08	< 0.001
Hypertension (Ref. No)							− 0.29 (− 1.13, 0.56)	0.01	0.51
Diabetes (Ref. No)							− 0.26 (− 1.38, 0.85)	0.01	0.64
Adjusted R^2^	0.22			0.22			0.22		

Model 1 was adjusted for sex, age, educational attainment, and mental health-related quality of life at baseline; Model 2 was additionally adjusted for body mass index, tobacco consumption, and physical activity; Model 3 was additionally adjusted for the presence of chronic conditions (cardiovascular diseases, neurological diseases, hypertension, and diabetes)

The relationship between loneliness and physical HRQoL was found to be different by sex (*p* for interaction term = 0.028) (Table 4). Women with higher levels of loneliness showed significantly lower scores in physical HRQoL [− 0.53 (− 0.92, − 0.14), *p* = 0.007], but this association was not found for men [0.07 (− 0.39, 0.54), *p* = 0.76]. On the other hand, the association between higher levels of loneliness and lower scores in mental HRQoL was significant in both men [− 0.77 (− 1.21, − 0.32), *p* < 0.001] and women [− 0.76 (− 1.25, -0.27), *p* = 0.002]. Significant associations were not found for social isolation in these stratified analyses.

**Table 4:** Stratified analysis, by sex, for the association between social isolation and loneliness, and physical and mental health-related quality of life, after the two-year follow-up period

	Men			Women			*p* for interaction
	Coef (95% CI)	|β|	*p*	Coef (95% CI)	|β|	*p*	
*Physical health-related quality of life*							
Social isolation	− 0.25 (− 0.88, 0.39)	0.02	0.45	0.44 (− 0.28, 1.17)	0.03	0.23	0.17
Loneliness	0.07 (− 0.39, 0.54)	0.01	0.76	− 0.53 (− 0.92, − 0.14)	0.08	0.007	0.028
Adjusted R^2^	0.22			0.35			
*Mental health-related quality of life*							
Social isolation	0.56 (− 0.03, 1.15)	0.06	0.06	0.07 (− 0.78, 0.92)	0.00	0.87	0.37
Loneliness	− 0.77 (− 1.21, − 0.32)	0.11	0.001	− 0.76 (− 1.25, − 0.27)	0.10	0.002	0.96
Adjusted R^2^	0.16			0.19			

All models were adjusted for age, educational attainment, body mass index, tobacco consumption, and physical activity, the presence of chronic conditions (cardiovascular diseases, neurological diseases, hypertension, and diabetes), and the corresponding SF-12 component (physical or mental health-related quality of life) at baseline

## Discussion

In our study population, after two years of follow-up, higher levels of loneliness were independently associated with lower mental and physical HRQoL scores. Social isolation did not show a significant association with either mental or physical HRQoL. The association between loneliness and mental HRQoL remained similar in the stratified analyses by sex, but a differential effect of sex was only found on the relation between loneliness and physical HRQoL, specifically in women. This study is one of the few that analyses the independent association between social isolation and loneliness, and HRQoL in both older men and women, using reliable measurement instruments. Even when there is a lot of research that combines the evaluation of loneliness and social isolation as a single concept, some other studies found a moderate or weak correlation among them when measured independently (Cornwell and Waite 2009; McHugh et al. 2017; Schrempft et al. 2019). Our study defined social isolation in terms of living in partnership, frequency of social interactions, and attending to civil or social organizations (Steptoe et al. 2013) and found a moderately low correlation with loneliness. Therefore, there is a need to assess loneliness and social isolation as separate concepts to better understand their impact on HRQoL as they encompass different aspects of social relationships; for this reason, the statistical models used in our study analyzed them simultaneously as independent variables to assess their differential association with physical and mental HRQoL.

Sex inequalities are often seen in older population, where women can feel a major weight in different activities, which can lead to a higher level of social isolation due to an increase in household activities and unpaid work such as caregiver roles (Boehlen et al. 2022; Gumà et al. 2019). Women in our cohort of Spanish older population may have had or experienced a higher burden in the household activities than men and less economic, social and labor market participation. Therefore, these social inequalities may be related to major risk of social isolation and loneliness in women (Gumà et al. 2019; Pérez-Hernández et al. 2017; United Nations 2023). In our study, we have found significantly higher levels of loneliness in women, but levels of social isolation were similar in both men and women.

Previous studies have found that lonely older people tend to present lower scores in both physical and mental HRQoL (Boehlen et al. 2022, 2023; Torres et al. 2024). Nonetheless, even when other studies agree on the relationship between loneliness and HRQoL, the potential differences by sex are not always evaluated since the prevalence of loneliness is considered to be similar in men and women (Leonti et al. 2025; Maes et al. 2019). The results found in our study add a novel perspective by showing a differential relationship by sex between loneliness and physical HRQoL, suggesting a significant relationship in women but not in men. These findings are consistent with those found by Boehlen et al. (2022), who also reported a significant association between loneliness and physical HRQoL in older women, using the SF-12 instrument in a German population. Sex differences could be explained by how women express and manifest their needs of existing and desired social relationships, since they tend to be more vulnerable to a lack of supportive relationships (Shin and Park 2023). By highlighting these sex differences, our study contributes to a more detailed understanding of this field of knowledge.

Although in our study we did not find a significant association between social isolation and physical and mental HRQoL, previous research has shown that socially isolated individuals reported lower HRQoL scores compared to those who were not socially isolated (Hawton et al. 2011; Sherman et al. 2024). These previous findings might be explained by changes in health-related lifestyle behaviors (e.g., smoking habits, alcohol consumption, and being physically active) aimed to belong to certain social groups (Kobayashi et al. 2018; Shankar et al. 2011). Golaszewski et al. (2022) suggested that older women tend to be more social isolated than men. In our study, significant differences were not found in social isolation by sex.

Some of the physiological mediators that may influence brain regulation under the presence of loneliness may act from the prefrontal cortex, which is largely connected to the limbic system, facilitating processes with other brain structures such as the amygdala and hippocampus (Cacioppo et al. 2015). These structures are known to mediate the regulation of emotions and behaviors, acting through the hypothalamic–pituitary adrenocortical (HPA) axis (Delgado et al. 2024; Watanasriyakul et al. 2019). Exposure to loneliness may trigger stressful situations and vice versa, and this can also activate the HPA axis (Stuller et al. 2012). Individuals experiencing loneliness are more likely to report poor physical health outcomes, including cardiovascular diseases, diabetes, and compromised immune function (Holt-Lunstad 2021; Leigh-Hunt et al. 2017; Lim et al. 2023). Also, this response could be affected during the aging process, due to a reduction in the vascularization to the central nervous system (Braveman and Gottlieb 2014; Cacioppo et al. 2015; Shankar et al. 2011). These mechanisms can explain why, in our study, higher levels of loneliness were associated with lower scores in mental and physical HRQoL.

The SF-12 health questionnaire used in our study to assess HRQoL is a subjective measure which includes mental health domains related to emotional problems and energy and feelings of fatigue, also associated with depressive symptoms. The significant differences in HRQoL found in our study between men and women could be also explained by the different expression of these symptoms by sex, since women are more likely to present and report symptoms related to depressed mood and somatic difficulties (Acciai and Hardy 2017; Cavanagh et al. 2017). The differential relationship found by sex between loneliness and physical HRQoL highlights the need of sex-specific interventions, particularly for women. The World Health Organization is promoting digital strategies to combat loneliness and social isolation (World Health Organization 2023b). Examples of strategies implemented include training in the use of digital tools to communicate better, access to tools that provide support to improve health, and the involvement of different members of the community. These strategies are addressed to different groups in the community, including people 60 years and older. Nonetheless, there is still a need for more evidence to demonstrate that digital interventions can effectively reduce loneliness and social isolation (Balki et al. 2022; Coll-Planas et al. 2024). Although loneliness and social isolation are increasingly on the international, national, and local political agenda, more steps are needed to move forward from knowledge to action on this issue in order to create interventions that are effective for the population (Goldman et al. 2024; Shah et al. 2021).

Among the strengths of this research, this is a cohort study conducted in older population where sociodemographic characteristics, lifestyle behaviors, social variables, and health-related outcomes are well characterized. The analytical sample has a moderately large size, where social isolation, loneliness, and HRQoL have been measured with specific and reliable instruments. Also, the analysis to explore the association between social variables and HRQoL have been adjusted for potential confounders, including sociodemographics, a wide set of lifestyle behaviors, and the presence of chronic conditions.

As potential limitations, the role of social isolation and other social variables in the relation remain to be disentangled and need to be assessed in further research. Other social variables such as social participation, social support, and quality of the social network could be measured and analyzed. The role of personality could be also considered in future studies, since introverted individuals might show high social isolation without presenting loneliness. Although the study has been conducted in an older population, the two-year follow-up period is moderately short and may make difficult to observe changes in HRQoL, underestimating the strength of some of the relationships evaluated; however, this follow-up period allows for considering a relatively large analytical sample, since the number of participants missed during the follow-up is lower. On the other hand, attrition bias could occur since the significant differences found in sociodemographic and clinical covariates between the analytical sample and those who did not participate in the follow-up period considered for the Seniors-ENRICA 2 cohort. Reverse causality cannot be discarded. Other prospective studies should confirm the results obtained, considering that data were taken in the years prior to the COVID-19 pandemic and could also serve as a reference for potential comparisons with recent data on social variables and HRQoL.

In conclusion, loneliness is inversely and independently related to physical and mental HRQoL, and these relations could be different by sex, after adjusting for potential confounders. Loneliness and social isolation are different concepts, and we have found different results in their association with HRQoL, although future studies should explore the independent effect of other social factors on both physical and mental components of HRQoL.

## Supplementary Information

Below is the link to the electronic supplementary material.

Supplementary file1 (DOCX 27 KB)

## Data Availability

Data used for the present study (including data collection methods and analytic code) are not openly available but will be available for other researchers upon reasonable request.
