# Supplementary material for: Loneliness, social isolation, and health-related quality of life in older adults
Source: Eur J Ageing. 2026 Feb 16;23(1):13. doi: 10.1007/s10433-025-00905-6 (PMC13013741; doi:10.1007/s10433-025-00905-6)
Supplement: Supplementary file 1 — Supplementary file1 (DOCX 27 KB) [file 10433_2025_905_MOESM1_ESM.docx]

**Title:** Loneliness, social isolation, and health-related quality of life in older adults.

**Author names:** Durán-Arias, Marcela^a^, MSc; Humberto Yévenes-Briones^a^, PhD; José R. Banegas^a,b^, MD, PhD; Fernando Rodríguez-Artalejo^a,b,c^, MD, PhD; Esther Lopez-Garcia^a,b,c^, PhD; Francisco Félix Caballero^a,b,*^, PhD.

**Author Affiliations:**

^a^Faculty of Medicine. Department of Preventive Medicine and Public Health. Universidad Autónoma de Madrid, Madrid, Spain.

^b^ CIBER of Epidemiology and Public Health (CIBERESP), Instituto de Salud Carlos III, Madrid, Spain.

^c^ IMDEA-Food Institute, CEI UAM+CSIC, Madrid, Spain.

***Corresponding Author:**

Francisco Félix Caballero, PhD

Department of Preventive Medicine and Public Health,

School of Medicine, Universidad Autónoma de Madrid,

Arzobispo Morcillo, 4, 28029, Madrid, Spain

Email: [felix.caballero@uam.es](mailto:felix.caballero@uam.es)

**Supplementary Table S1.** SF-12 Health Survey with the different answer options and weights used to obtain the physical and mental HRQoL scores.

| **SF-12 question** | **Answer options** | **Physical weight** | **Mental weight** |
| --- | --- | --- | --- |
| 1. In general, would you say your health is:  (Reference: Excellent) | Very Good | -0.93 | 0.371 |
|  | Good | -2.355 | 0.459 |
|  | Fair | -5.129 | 0.863 |
|  | Poor | -8.491 | 1.203 |
| 2. Does your health now limit you in moderate activities (e.g., moving a table, pushing a vacuum cleaner, bowling)?  (Reference: No, it does not) | Yes, limited a lot | -5.463 | 2.336 |
|  | Yes, limited a little | -3.161 | 1.472 |
| 3. Does your health now limit you in climbing several flights of stairs?  (Reference: No, it does not) | Yes, limited a lot | -7.36 | 3.272 |
|  | Yes, limited a little | -3.383 | 1.565 |
| During the past 4 weeks: |  |  |  |
| 4. Have you accomplished less than you would like as a result of your physical health?  (Reference: No) | Yes | -4.549 | 1.279 |
| 5. Were you limited in the kind of work or other activities as a result of your physical health?  (Reference: No) | Yes | -5.93 | 2.86 |
| 6. Have you accomplished less than you would like as a result of any emotional problems (such as feeling depressed or anxious)?  (Reference: No) | Yes | 4.043 | -9.719 |
| 7. Didn´t do work or other activities as carefully as usual, as a result of any emotional problems (such as feeling depressed or anxious)?  (Reference: No) | Yes | 3.468 | -8.052 |
| 8. How much did pain interfere with your normal work (including work outside the home and housework)?  (Reference: Not at all) | A little bit | -4.236 | 1.383 |
|  | Moderately | -6.356 | 1.481 |
|  | Quite a bit | -8.242 | 2.439 |
|  | Extremely | -10.215 | 2.256 |
| 9. How much of the time have you felt calm and peaceful?  (Reference: All of the Time) | Most of the Time | 0.372 | -1.853 |
|  | Some of the Time | 1.903 | -5.87 |
|  | A Little of the Time | 2.401 | -7.563 |
|  | None of the Time/Never | 3.081 | -9.374 |
| 10. Did you have a lot of energy?  (Reference: All the Time) | Most of the Time | -0.13 | -1.018 |
|  | Some of the Time | -0.795 | -3.039 |
|  | A Little of the Time | -1.183 | -3.997 |
|  | None of the Time/Never | -1.374 | -5.454 |
| 11. Have you felt downhearted and blue?  (Reference: None of the Time) | All of the Time | 3.838 | -12.617 |
|  | Most of the Time | 2.809 | -9.942 |
|  | Some of the Time | 1.087 | -4.773 |
|  | A Little of the Time | 0.589 | -2.468 |
| 12. How much of the time has your physical health or emotional problems interfered with your social activities (like visiting friends, relatives, etc.)?  (Reference: None of the Time) | All of the Time | -1.82 | -10.386 |
|  | Most of the Time | -1.175 | -7.652 |
|  | Some of the Time | -0.632 | -5.033 |
|  | A Little of the Time | -0.396 | -2.63 |

Note: HRQoL = Health-related quality of life; Physical and mental weights are taken from Vilagut et al. (2008). They obtained these weights in a Spanish sample which was representative of the Spanish adult population.

**Supplementary Table S2.** Sociodemographic and clinical characteristics for subjects included in the analytical sample and subjects who did not participate in the follow-up phase.

|  | **Analytical sample**  n = 1808 | **Subjects who did not participate in the follow-up**  n = 1379 | ***p**** |
| --- | --- | --- | --- |
| Physical HRQoL, Mean ±SD | 51.1 ± 9.4 | 48.5 ± 10.6 | <0.001 |
| Mental HRQoL, Mean ± SD | 50.9 ± 9.1 | 48.9 ± 10.8 | <0.001 |
| Social isolation, Mean ± SD | 0.8 ± 0.8 | 0.9 ± 0.8 | 0.004 |
| Loneliness, Mean ± SD | 3.7 ± 1.4 | 3.8 ± 1.5 | 0.09 |
| Women, n (%) | 923 (51.1) | 771 (55.9) | 0.006 |
| Age (years), Mean ± SD | 71.4 ± 4.2 | 72.4 ± 4.7 | <0.001 |
| Educational attainment, n (%) |  |  | <0.001 |
| Primary school or lower | 1081 (59.8) | 972 (70.5) |  |
| Secondary school | 348 (19.3) | 242 (17.6) |  |
| University studies | 379 (21.0) | 165 (12.0) |  |
| Body mass index, n (%) |  |  | 0.045 |
| <25 kg/m^2^ | 487 (26.9) | 231 (24.7) |  |
| 25-29.9 kg/m^2^ | 859 (47.5) | 424 (45.4) |  |
| ≥ 30 kg/m^2^ | 462 (25.6) | 280 (30.0) |  |
| Smoking status, n (%) |  |  | 0.11 |
| Never smoker | 930 (51.4) | 752 (54.5) |  |
| Former smoker | 714 (39.5) | 494 (35.8) |  |
| Current smoker | 164 (9.1) | 133 (9.6) |  |
| Chronic conditions, n (%) |  |  |  |
| Cardiovascular diseases | 117 (6.5) | 100 (7.3) | 0.35 |
| Neurological diseases | 217 (12.0) | 191 (13.9) | 0.12 |
| Hypertension | 993 (54.9) | 806 (58.8) | 0.029 |
| Diabetes | 305 (16.9) | 295 (21.5) | 0.001 |

Note. SD = Standard deviation; HRQoL = Health-related quality of life. * Unpaired t-test was used for comparing means in quantitative variables, while chi-square test was used for comparing percentages in categorical ones.
